# In-Vitro and In-Vivo Evaluation of Velpatasvir- Loaded Mesoporous Silica Scaffolds. A Prospective Carrier for Drug Bioavailability Enhancement

**DOI:** 10.3390/pharmaceutics12040307

**Published:** 2020-03-28

**Authors:** Yasir Mehmood, Ikram Ullah Khan, Yasser Shahzad, Rizwan Ullah Khan, Muhammad Shahid Iqbal, Haseeb Ahmad Khan, Ikrima Khalid, Abid Mehmood Yousaf, Syed Haroon Khalid, Sajid Asghar, Muhammad Asif, Talib Hussain, Shefaat Ullah Shah

**Affiliations:** 1 Department of Pharmaceutics, Faculty of Pharmaceutical Sciences, Government College University Faisalabad, Faisalabad 38000, Pakistan; 2 Department of Pharmacy, COMSATS University Islamabad, Lahore Campus, Lahore 54000, Pakistan; 3 Department of Pathology, Prince Faisal Cancer Centre, Buraydah Al Qassim 51431, Saudi Arabia; 4 Department of Clinical Pharmacy, College of Pharmacy, Prince Sattam bin Abdulaziz University, Alkharj 11492, Saudi Arabia; 5 Department of Pathology, FMH College of Medicine and Dentistry, Lahore 54000, Pakistan; 6 Department of Pharmacology, Faculty of Pharmaceutical Sciences, Government College University Faisalabad, Faisalabad 38000, Pakistan; 7 Department of Pharmacy, The Islamia University of Bahawalpur, Bahawalpur 63100, Pakistan; 8 Department of Pharmaceutics, Faculty of Pharmacy, Gomal University, Dera Ismail Khan 29050, Pakistan

**Keywords:** velpatasvir, solubility, bioavailability, mesoporous silica nanoparticles

## Abstract

The limited aqueous solubility of many active pharmaceutical ingredients (APIs) is responsible for their poor performance and low drug levels in blood and at target sites. Various approaches have been adopted to tackle this issue. Most recently, mesoporous silica nanoparticles (MSN) have gained attention of pharmaceutical scientists for bio-imaging, bio-sensing, gene delivery, drug solubility enhancement, and controlled and targeted drug release. Here, we have successfully incorporated the poorly water soluble antiviral drug velpatasvir (VLP) in MSN. These spherical particles were 186 nm in diameter with polydispersity index of 0.244. Blank MSN have specific surface area and pore diameter of 602.5 ± 0.7 m^2^/g and 5.9 nm, respectively, which reduced after successful incorporation of drug. Drug was in amorphous form in synthesized VLP-loaded silica particles (VLP-MSN) with no significant interaction with carrier. Pure VLP showed poor dissolution with progressive increment in pH of dissolution media which could limit its availability in systemic circulation after oral administration. After VLP loading in silica carriers, drug released rapidly over a wide range of pH values, i.e., 1.2 to 6.8, thus indicating an improvement in the solubility profile of VLP. These particles were biocompatible, with an LD_50_ of 448 µg/mL, and *in-vivo* pharmacokinetic results demonstrated that VLP-MSN significantly enhanced the bioavailability as compared to pure drug. The above results clearly demonstrate satisfactory *in-vitro* performance, biocompatibility, non-toxicity and *in-vivo* bioavailability enhancement with VLP-MSN.

## 1. Introduction

In the modern era approximately 40% of marketed oral drugs are deemed practically insoluble or poorly soluble [1]. Poorly soluble drugs cause many issues which include dose escalation, low bioavailability, and poor *in-vitro* and *in-vivo* drug performance [2]. To curtail this issue, scientists have adopted various approaches, for example micronization, complexation, lipid-based formulations, co-solvents, salt formation, solid dispersions, nano-formulations and many more [3,4]. Among the above-mentioned techniques, nanocarriers, which include drug nanocrystals [5,6,7], nanoemulsions [8,9,10], polymeric micelles [11,12], nanoparticles [13,14], nanotubes [13], and silica-based nanocarriers [15] are widely investigated by scientists.

In the recent past, scientists have shown keen interest in mesoporous silica nanoparticles (MSNs), since they possess attractive features such as biocompatibility, non-toxicity, effective cellular uptake, possibility for drug loading and manageable drug discharge [16,17]. These favourable features make MSNs promising contenders for numerous applications, including drug delivery [3,18], theranostic [19,20], biocatalysis [21,22] and tissue engineering [23,24]. Likewise, one of the primary benefits of these materials is the observed improvement in oral bioavailability of low water soluble medicines [25]. In MSNs, particle morphology [26], pore size [27,28,29,30,31], surface area [32], particle size, surface coating [33,34], loading method [35,36,37,38] and drug/excipient ratio are the key features which control drug dissolution.

One of the main features needed to move forward with MSN utilization in medicine and pharmaceuticals would be learning how to control MSN pore size. MSNs can only loads into their pores molecules whose molecular dimensions are less than the pore diameter (<10 nm) [39]. Therefore, it is very important to consider the pore size of MSNs and molecular dimensions while choosing a drug for encapsulation.

Velpatasvir (VLP) is a direct-acting antiviral agent (DAA) against hepatitis C virus (HCV) [40]. VLP is a novel HCV NS5A inhibitor with potent antiviral activity against genotype 1 to 6 replicons. VLP is defined as a Biopharmaceutical Classification System (BCS) class IV drug, with low aqueous solubility and low permeability. It is soluble at pH 1.2, sparingly soluble at pH 2, and practically insoluble at pH > 5 [40,41]. This behaviour of the drug is responsible for its poor bioavailability as it is reported that in a fasting state, the pH of the stomach lies between 1.3 to 2.5 and raises to 4.5 to 5.8 within an hour after eating [42]. Furthermore, even a glass of water can also immediately raise the intragastric pH > 4 [43]. Therefore, there is a need to develop effective methods to enhance the solubility, permeability and bioavailability of VLP. In order to resolve the aforementioned questions, we have designed VLP-loaded mesoporous nanoparticles for the very first time and extensively characterized them by various *in-vitro* and *in-vivo* techniques.

## 2. Materials and Methods

### 2.1. Materials 

Cetyltrimethyl-ammonium bromide (CTAB, 99.0%) as surfactant and the alkoxide precursor tetraethyl orthosilicate (TEOS, 99.0%) were purchased from Dae-Jung Chemicals (Siheung-si, Korea). VLP was generously donated by Ameer & Adnan Pharmaceutical Pvt. Ltd. (Lahore, Pakistan). Acetonitrile (≥98%) was also purchased from Dae-Jung Chemicals (Siheung-si, Korea). The rest of the chemicals used in this research were of analytical grade and used as such.

### 2.2. Methods

#### 2.2.1. Preparation of VLP-MSNs

MSNs were fabricated by following a sol-gel method in an oil/water phase, as reported by [44,45] with a few minor changes. The sol-gel was prepared by dissolving 500 mg of CTAB in mixture of water and acetonitrile (1:1) at 35 °C and pH was adjusted to 9.0 with 0.5 M KOH. The solution was constantly stirred at 1500 rpm and a nitrogen flow was maintained throughout the reaction. When a clear solution was obtained, a defined quantity of TEOS (5 mL) was added drop by drop to the resultant solution. After a few minutes, the solution turned opaque, indicating the start of the reaction and a gel was formed. The white gel was filtered using a 0.1 μm filter (Sartorius, Goettingen, Germany) under nitrogen pressure and the gel washed with deionized water. The gel was converted into a fine powder and then dried overnight at room temperature. To completely remove the surfactant, the powder was calcined at 500 °C for 6 h and named as MSN.

VLP was loaded in nanocarriers by adsorption, which includes stirring and soaking as reported previously [46]. Concentrated solution (1 mg/mL) of VLP was obtained in methanol. Afterwards, MSNs were mixed with VLP in a drug-silica ratio of 1:1 (*w/w*). This mixture was stirred continuously for three hours and later kept soaked for three days. Thereafter, particles were filtered by using a membrane filter (0.1 µm), and washed thrice with 100 mL of methanol to remove any surface-adhered VLP. Samples were dried overnight at room temperature and then further dried at 50 °C under vacuum. These loaded particles were designated as VLP-MSN.

#### 2.2.2. Drug Loading and Entrapment Efficiency

To determine the VLP-MSN contents, 100 mg of particles were stirred in 100 mL of methanol for one hour. Extracted VLP was quantified by calculating area under the curve using a LC 20A RP-HPLC system consisting of a 1000 pump and 2500 UV-VIS detector (Shimadzu, KS, USA) and an Agela C18 column (250×4.6 mm, 5 µm)) with detection at 270 nm. The mobile phase was composed of ammonium acetate buffer, methanol and acetonitrile in the ratio of 20:40:40 (*v/v*) and its flow was maintained at 1 mL/min. Each time 20 µL of analyte was injected and obtained peaks were compared with the standard (*n* = 3). Drug loading and efficiency was calculated as follows:

Loading capacity = $\frac{\mathrm{Entrapped}\;\mathrm{Drug}}{\mathrm{Total}\;\mathrm{weight}\;\mathrm{of}\;\mathrm{nanoparticles}}\times 100$

Entrapment efficiency (%) = $\frac{\mathrm{Drugadded}-\mathrm{Freedrug}}{\mathrm{Drugadded}}\times \;100$

#### 2.2.3. Scanning Electron Microscopy (SEM)

Shape and surface morphology of blank and drug loaded MSN were investigated by SEM (VEGA3 TESCAN, Brno – kohoutovice, Czech Republic) after gold coating [34,47].

#### 2.2.4. Dynamic Light Scattering (DLS)

Particle size and zeta potential of MSN was measured at 25 °C using water as dispersant [48] employing a Zetasizer Nano ZS instrument (Malvern Panalytical Ltd, Malvern, UK). 

#### 2.2.5. Transmission Electron Microscopy (TEM)

TEM images were obtained on a JEM 2100F analytical electron microscope (JEOL, Tokyo, Japan) having a field emission electron gun operating at 200 kV.

#### 2.2.6. Nitrogen-Adsorption/Desorption

Specific surface area (B_SSA_), pore volume and size of blank and VLP loaded MSN were calculated by N_2_ adsorption/desorption analysis (Gemini VII 2390 surface area analyzer, Micromeritics, Norcross, USA) operating at −196.15 °C. Both samples were degassed at 200 °C for 24 h prior to analysis. Barrett-Joyner-Halenda (BJH) model was applied to determine pore size and pore volume distribution from desorption isotherm. The B_SSA_ was calculated through Brunauer-Emmett-Teller (BET) using adsorption data at relative pressure (p/p°) [47].

#### 2.2.7. Fourier Transform Infra-Red Spectroscopy (FTIR)

FTIR spectra of VLP, blank and drug-loaded MSN were obtained by using a Nicolet IS7ATR-FTIR spectrometer FTIR spectrometer (Thermo Scientific, Waltham, MA, USA). FTIR spectra were obtained over the spectral range of 500–4000 cm^−1^ and at a 2 cm^−1^ resolution [49].

#### 2.2.8. Powder X-ray Diffraction (XRD)

To investigate the influence of processing parameters on VLP loading, X-ray diffraction patterns of VLP and silica particles were obtained using an X-Ray diffractometer (X’pert PRO, PANalytical, Almelo, Netherlands) using a Cu Kα radiation source which operates at 30 mA and 30 kV. All data were recorded from 2θ angle of 5° to 40° at a step size of 0.02° and scanning speed of 4°/min [47,48].

#### 2.2.9. Thermal Analysis

DSC and TGA analysis of the VLP, blank and drug loaded MSN were carried out in a Q1000 system (TA Instruments, New Castle, DE, USA) using a heating rate of 10 °C/min under a nitrogen purge of 40 mL/min [34,47].

#### 2.2.10. In-Vitro Dissolution

*In-vitro* dissolution was used to investigate release profile of VLP loaded MSN. Conditions were maintained as described in food and drug administration (FDA) i.e., 75 ± 1 rpm and 37 °C VLP-MSN equivalent to 100 mg VLP was exposed to two different dissolution medium with pH 1.2 and 6.8 in USP type II apparatus (Model- DT 120, Cure Apparatus, Lahore, Pakistan) for 180 min. At predetermined time intervals, samples were withdrawn from dissolution medium and same volume was replaced. Concentration of VLP was calculated from previously constructed calibration curve [34].

#### 2.2.11. Viability of Cells

The viability of cells were investigated by using 3-[4,5-dimethylthialzol-2-yl]-2,5-diphenyltetrazolium bromide (MTT, Sigma, St Louis, MO, USA) assay. Here, HEP G2 cells were seeded in 96-well plates at a density of 1 × 10^4^ per well in 100 μL of media and grown overnight. The cells were incubated with VLP loaded MSN for 24 h. The absorbance was measured at 570 nm by multi-detection microplate reader (Synergy^TM^ HT, BioTek Instruments Inc, Winooski, VT, USA). We carried out experiments in triplicate and results were expressed as a percentage of viable cells in treated and the control group [50].

#### 2.2.12. Toxicological Evaluation

In order to gain insight into *in-vivo* toxicity profile of our formulation, fifteen male Sprague-Dawley rats (200–210 g weight, age 6–8 weeks) were obtained from the in-house animal facility. These animals were kept in standard laboratory conditions i.e., in open-mesh stainless steel cages with radiation sterilized paddy husk for bedding and were housed at 12-h artificial light/dark cycles, 25 °C ± 1 °C and 60 ± 10% humidity. Before experimentation, animals were acclimatized in these condition for one week with free accesses to food and water ad libitum. After one week, rats were divided into three groups of five rats each. Firsts group was control and did not received any treatment. Second group of rats received VLP (1.4 mg/kg) and third group received VLP-MSN (VLP-MSN equivalent to 1.4 mg of VLP) admixed with food at the start of the experiment. These animals were regularly checked for behavioural and weight changes at day 1, 5, 10 and 14. We strictly followed Organization of Economic Co-operation and Development (OECD) guidelines and all of experimental protocols were by the institutional Ethical Review Committee (ERC) of Government College University Faisalabad, Faisalabad Pakistan (Reference Number: GCUF/ERC/2041, Dated 05/07/2019). 

We assessed various biochemical, haematological, and histological parameters to evaluate toxicity profile in rats. For biochemical and haematological assessment, blood samples were collected in EDTA tubes on 14^th^ day of experiment by cardiac puncture and later used for analysis. Animals were sacrificed at end of study and vital organs such as heart, lung, liver, and kidneys were isolated and fixed with 10% neutral buffered formalin, and later on histological analysis was performed after hematoxylin and eosin staining.

#### 2.2.13. Pharmacokinetic Study

Pharmacokinetic studies were performed for pure VLP and VLP loaded mesoporous silica in Sprague- Dawley rats. These animals weighed more than 200 g and were kept in cages under controlled conditions (*n* = 6) with free access to water and feed. They were kept under fasting conditions before the experiments. Pure VLP (1.4 mg/Kg) and VLP-MSN (VLP-MSN equivalent to 1.4 mg of VLP) were given orally to rats and later blood samples (300 µL) were taken in heparinised tubes after a specific time interval via the femoral artery. Plasma was separated by centrifugation (4000 rpm, 15 min) and stored in Eppendorf tubes at −20 °C. Drug was extracted by liquid-liquid extraction. 100 µL of plasma was spiked with 10 µL of internal standard (tramadol HCl, 10 µg/mL) and diluted with 890 µL acetonitrile. Mixture was vortexed for 5 min and then centrifuged at 4000 rpm for 15 min to separate the supernatant organic solvent. The separated layer was dried in an oven. Afterwards, the dried residue was reconstituted with 100 µL of mobile phase (methanol, phosphoric acid buffer and acetonitrile (40: 20:40 *v/v*)), vortexed and injected (20 µL) into the RP-HPLC system at a flow rate of 1 mL/min using a Promosil C18 column (4.6 × 250 mm, 5 µm). Pharmacokinetic parameters were determined by using PK solver software. Pharmacokinetic study was approved by the Ethics Committee, Government college university Faisalabad, Pakistan (Study No: 19641).

## 3. Results and Discussion

Here we developed MSN particles by sol-gel process as reported previously [45]. This method gave us a good yield (96%) of silica particles that were loaded with VLP (encapsulation efficiency 27%, 0.275 mg of VLP is loaded per mg of particles). Later on these particles were subjected to further in-vitro and in-vivo characterization.

### 3.1. Microscopic Analysis

To verify the ordered mesoporous structure of MSN, TEM was employed. The TEM images indicated a porous structure and ordered 2D hexagonal structure was later confirmed by specific XRD peaks. Chaing et al. prepared MSN particles in aqueous media and reported an influence of silica source, pH and reaction time on their structure and size. They obtained uniform size particles with an ordered structure at high pH (12 or 13) and non-ordered at low pH 9.5 [51], but we obtained ordered particles at pH 9, which could be possibly due to change of solvent which in our case was water:acetronitrile in equal ratio under basic conditions.

**Figure 1:**
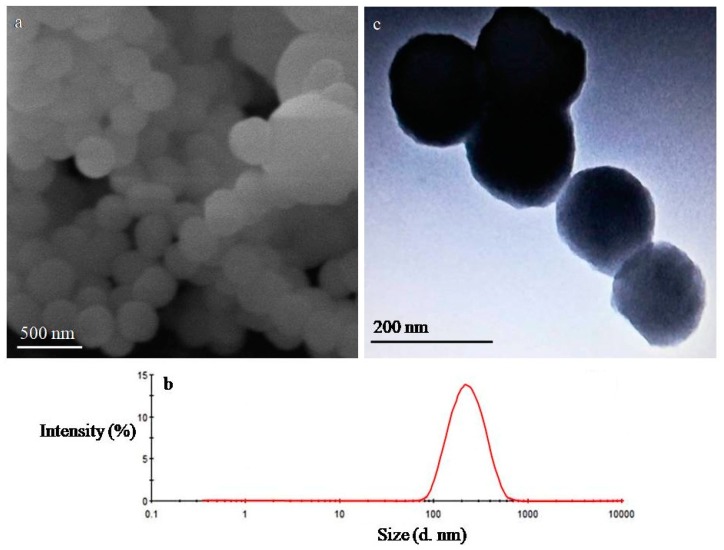
SEM (**a**), particle size distribution (**b**) of VLP-MSN and TEM (**c**) images of MSN respectively.

Similar results were reported in another study where ordered silica particles were obtained at pH 10 [52]. The morphology of VLP-MSN particles was analysed by SEM and TEM. SEM images showed the VLP-MSNs were spherical in shape and uniform in size as shown in Figure 1a and later confirmed by DLS results (Figure 1b).

### 3.2. Dynamic Light Scattering

We employed DLS to determine particle size, size distribution and zeta potential of the VLP-MSNs as explained by Powers et al. [53]. The average particle size, PDI and zeta potential of the VLP-MSNs was 186 nm, 0.244 (Figure 1b) and -8.39 mV, respectively. Low PDI of drug-loaded particles indicated a uniform size [54] and further substantiates our SEM results as mentioned in the section above.

Zeta potential also helps to predict surface charge, stability and possible cellular interaction of VLP loaded particles [55]. Nanocarriers with cationic charge react readily with cellular membranes as compared to ones with negative charge [56]. Furthermore, charges on the particles prevent their aggregation and hence enhances their stability [57]. Thus optimum size, PDI and charge make VLP-MSN suitable for drug delivery with minimum toxicity to biological membranes.

### 3.3. Nitrogen Adsorption/Desorption Studies

The nitrogen adsorption desorption method was employed to determine B_SSA_, pore volume and pore size distribution of the MSNs and VLP-MSNs. B_SSA_, pore volume and pore size of the MSNs before drug loading was 602.5 ± 0.7 m^2^/g, 0.946 ± 0.045 cm^3^/g and 5.9 ± 0.3 nm respectively.

**Figure 2:**
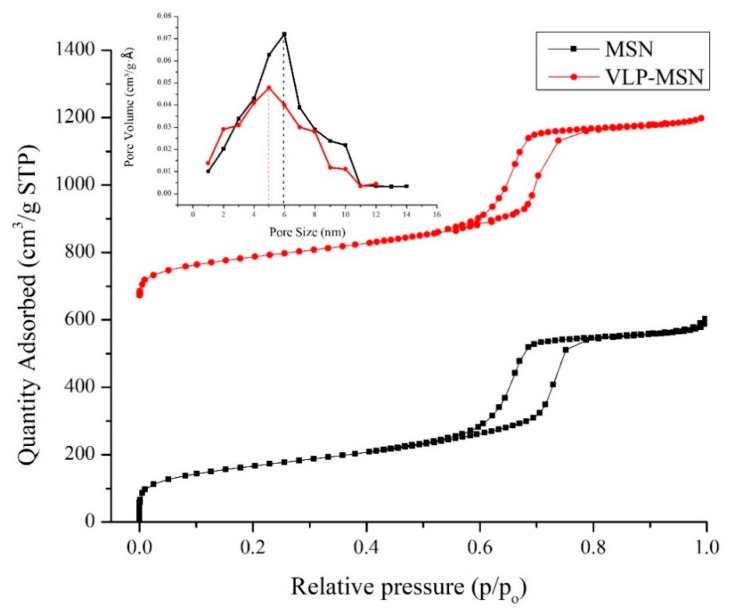
N_2_ adsorption-desorption isotherms of MSN and VLP-MSN. Inset picture shows pore diameter of MSN and VLP-MSN.

The data exhibited type IV isotherms (Figure 2) which are associated with mesoporous materials as per the IUPAC classification of porous materials. The hysteresis loops (two branches of the isotherms) obtained at relative pressures of 0.6 to 0.8 are almost vertical and nearly parallel during nitrogen gas uptake, and thus are referred to as H1 hysteresis loops employing regular mesoporous channels [45,52]. These parameters indicates mesoporous nature of particles as explained by [45,58,59]. Furthermore, this high B_SSA_ and pore volume indicates its potential to host drug molecules for various drug delivery application.

After loading of antiviral drug B_SSA,_ pore volume and pore size were reduced to 583.2 ± 4.8 m^2^/g, 0.893 ± 0.029 and 5 ± 0.2 nm, respectively, as shown in Figure 2. This decrease was attributed to loading of VLP in pores. Many other studies support our findings, for example Zhang et al. loaded telmisartan in silica based nanoparticles and observed reduction in pore size and B_SSA_ [34].

### 3.4. Fourier-Transform Infrared Spectroscopy 

**Figure 3:**
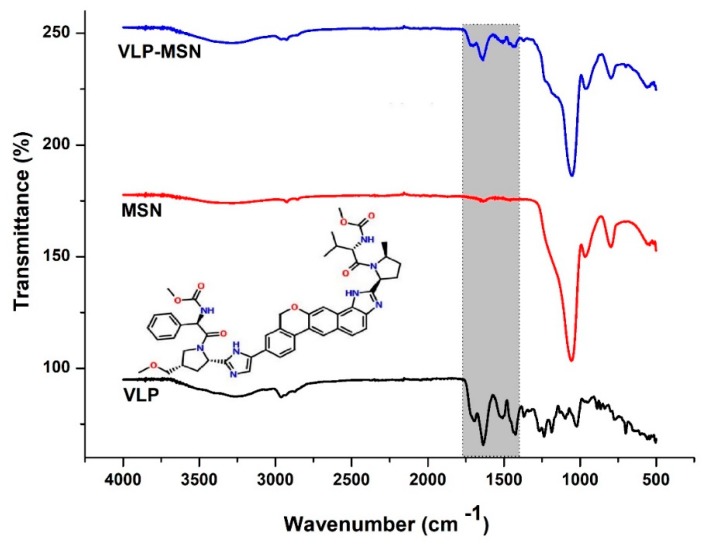
FTIR spectra of MSNs, VLP and VLP-MSNs. Inset picture shows the chemical structure of VLP while the shadowed region shows VLP peaks in pure sample and drug loaded particles.

FTIR studies not only provide information about different chemical groups but also about possible drug-carrier interactions. Figure 3 shows the FTIR spectra of VLP, MSNs and VLP-MSNs. MSNs showed absorption bands of the silicate at 797.97, 1053.78, 1636.88, 3396.49 cm^−1^. In the MSN case, a peak at 797.97 cm^−1^ indicated the absorption bands of the silicate and a peak at 1053.78 cm^−1^ showed the Si–O–Si bending [60,61]. A broader peak at 3396.49 cm^−1^ indicated silanol (Si–OH) symmetric stretching [62] and bending vibrations at 1636 cm^−1^ [45]. VLP showed main peaks at 1236.23, 1424.06, 1508.11, 1636.33, 1696.03, and 2961.38 cm^−1^. Peaks at 2961.38 and 1508.11 cm^−1^ indicated stretching vibration due to C–H band and C=C–C aromatic ring stretching, respectively. Similarly, characteristic peak at 1636.33 cm^−1^ depicted N–H bending vibration. Furthermore, peaks at 1424.06 and 1696.03 cm^−1^ correspond to the C–H asymmetric stretching and C=C stretching, respectively. 

FTIR spectrum of MSM-VLPs showed a characteristic peak of Si–O–Si bending at 1053 cm^−1^ as revealed by the MSN spectrum. Similarly, MSM-VLPs showed characteristic peaks at 1637.20, 1507.89 and 1438.09 cm^−1^ due to N–H bending vibrations, C=C–C aromatic ring stretching and C–H asymmetric stretching, respectively, which indicated the presence of drug in the formed VLP-MSNs without any prominent interactions.

### 3.5. X-ray Powder Diffraction 

**Figure 4:**
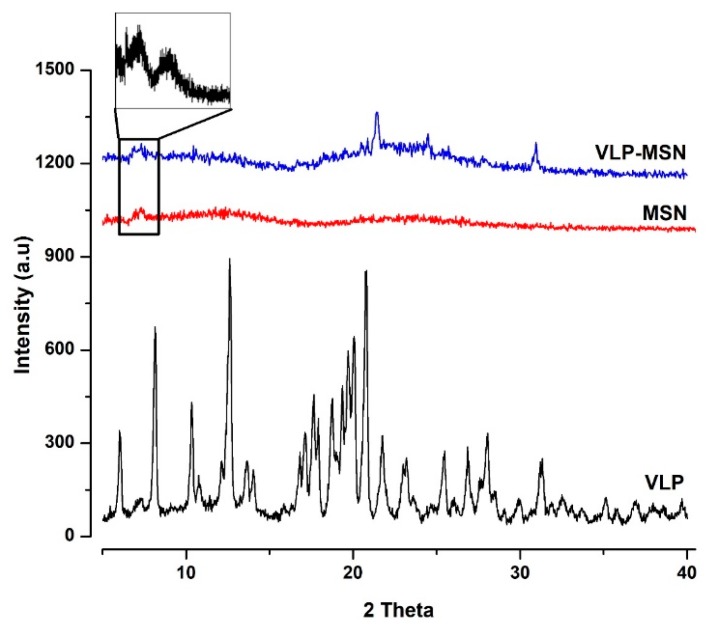
XRD pattern of VLP, MSNs and VLP-MSNs. The inset picture shows diffraction peaks of silica- based nanoparticles.

XRD analysis was used to investigate crystalline and amorphous state of the drug in blank and VLP-MSNs [63] and are shown in Figure 4. Pure VLP showed sharp diffraction peaks indicating its crystalline nature. We observed six distinct peaks for drug at 6.0°, 8.0°, 10.0°, 12.0°, 21.0°and 31.0^o^. Furthermore, in pure silica nanoparticles we observed distinct peaks which are possible indication of ordered 2D hexagonal mesoporous structure and two broad diffusion peaks recorded around 10^o^ and 23^o^ gives indication of their amorphous nature as mentioned in the literature [45,51,64].

In VLP-MSNs we observed a dramatic reduction in the characteristic peaks of VLP which could possibly be due to ionic interactions between silanol group of silica and the amine groups of VLP molecules thus leading to disordering of the crystalline structure when encapsulated in the MSN pores. Zhang et al. entrapped telmisartan in silica nanoparticles and reported a change of the crystalline drug to an amorphous form due to interactions between silica silanol groups and the carboxyl groups of the drug [34].

### 3.6. Thermogravimetric Analysis 

**Figure 5:**
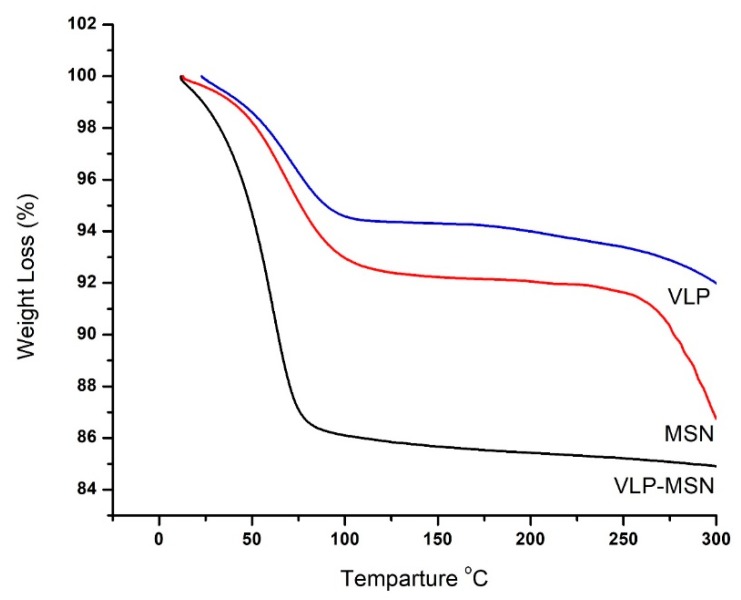
TGA thermograms of VLP, MSN and VLP-MSN.

Figure 5 shows TGA thermograms of VLP, MSNs and VLP-MSNs. MSNs show a 7% weight loss from 100 to 94 °C, corresponding to a water loss with increase in temperature. Further decrease in weight starts at this temperature and continues till complete degradation of formulation.

The TGA thermogram of VLP shows an initial 6% degradation which lasts up to 100 °C, followed by a 4% weight loss which occurs in the range of 100–350 °C indicating dehydration and deamination of the drug, respectively. Final degradation starts at 350 °C and lasts up to the complete degradation of the drug. From the VLP-MSN thermogram, it is seen that an initial weight loss of 15% falls in the temperature range of 0–78 °C revealing the initial dehydration of polymer chains. However, with increasing temperature up to 300^o^C, no further decomposition was found in VLP-MSNs.

### 3.7. Differential Scanning Calorimetry 

**Figure 6:**
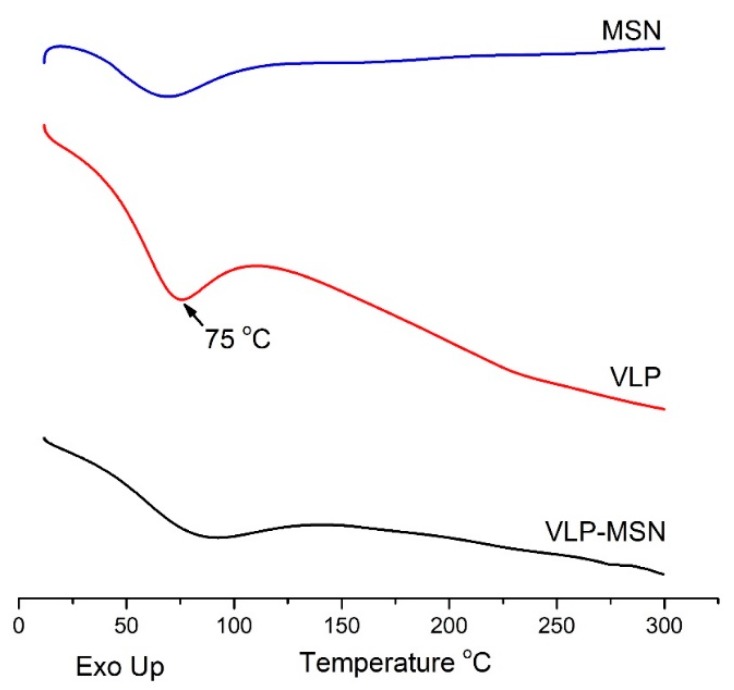
DSC thermograms of MSN, VLP, and VLP-MSN.

A DSC study was conducted to investigate VLP entrapment in the pores of the MSN particles. The presence or absence of crystalline drug was also confirmed by DSC analysis. When in its crystalline form in the pores, the amount of drug can be distinguished and estimated from the melting point depression using DSC. If the drug in the pores is in a non-crystalline state, no melting point depression can be detected [65,66]. As shown in Figure 6, the DSC curve of MSN exhibited an endothermic phase transition at 53 °C which corresponds to its glass transition temperature (Tg) where the amorphous sample went through a transition to a rubbery state as reported in the literature for porous silica nanoparticles [64]. 

The DSC curve for VLP reveals an endothermic peak at 30 °C which may be attributed to dehydration while an endothermic peak at about 75 °C reveals its melting point. In VLP-MSNs, no endothermic peak was observed at 75 °C, indicating the non-crystalline state of the velpatasvir. However, a weak and broad endothermic peak appears at 90 °C in VLP-MSNs which indicates a non-crystalline state of the drug entrapped in the MSNs. The melting peak depression of the VLP-MSNs may be attributed to the partial interaction of drug with the surface of the mesoporous nanoparticles which are rich in silica groups, causing partial amorphization of the VLP drug [67]. DSC results further confirms our XRD results. Similarly, the glass transition temperature for VLP-MSNs is slightly shifted to higher values as compared to pure MSNs, which is possibly due to the inclusion of VLP in the porous structure. Thus, DSC and TGA also provided evidence of complete inclusion of the drug into MSNs [68].

### 3.8. In-Vitro Release Studies

**Figure 7:**
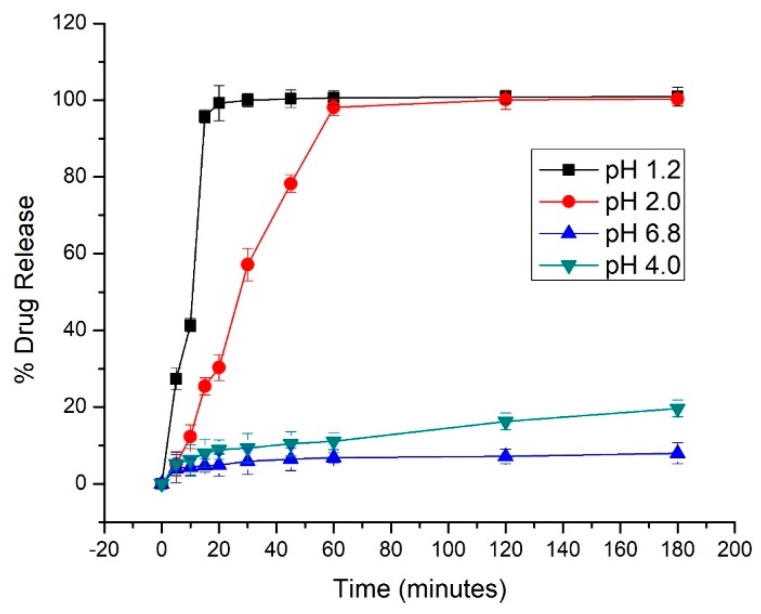
Release profiles of VLP in buffer (pH 1.2, 2, 4 & 6.8). Error bars indicate the standard deviation (*n* = 3).

We compared the dissolution profiles of pure drug in various buffer solutions. Pure VLP showed a pH dependent release, where it showed good dissolution up to pH of 2, after which it decreased drastically as shown in Figure 7. This could possibly be due to the low solubility of VLP at higher pH values [40,41,69,70,71].

**Figure 8:**
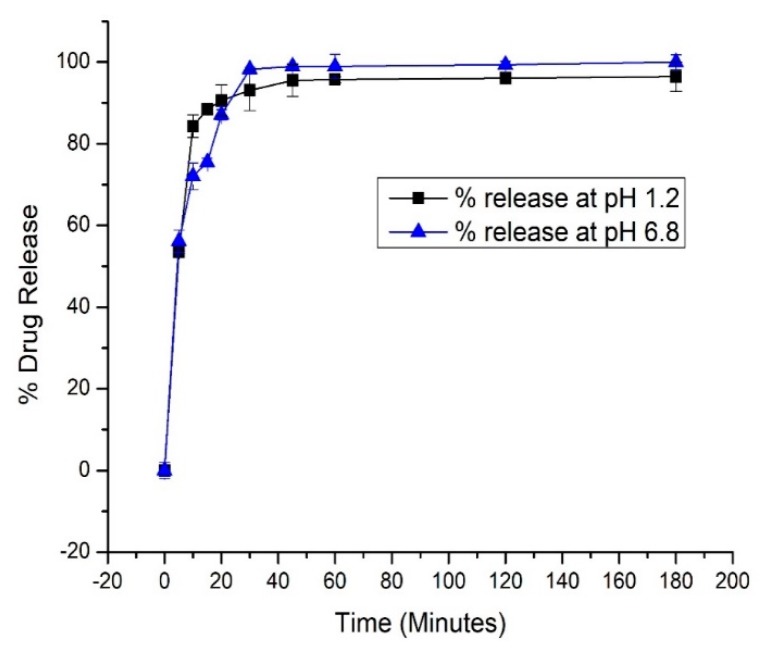
Release profiles of VLP-MSN in buffer (pH 1.2, 6.8). Error bars indicate the standard deviation (*n* = 3).

Our developed formulation, i.e., VLP-MSN, released 53% (at pH 1.2, it showed no significant difference from VLP) and 56% (at pH 6.8) of the drug during the first five minutes and more than 90% within 45 min. Thus, in drug release medium of pH 6.8, MSN acts as a carrier and boosts the dissolution profile of VLP as shown in Figure 8. This dissolution enhancement is attributed to different factors such as: (a) presence of drug in amorphous form due to its interaction with the carrier, (b) presence of drug in confined channels reduces the drug particle size to nanometer range, suppresses the crystallization of VLP and makes the VLP more soluble, (c) lastly the large surface area and pore size facilitates the diffusion of the drug [65,67,72,73]. Thus, these multiple factors contribute to enhance the dissolution rate of the entrapped drug. As our particles lead to solubility enhancement at higher pH these developed VLP-MSNs could overcome the pH-dependent bioavailability issues related with oral administration of VLP.

### 3.9. Cytotoxicity Assay

**Figure 9:**
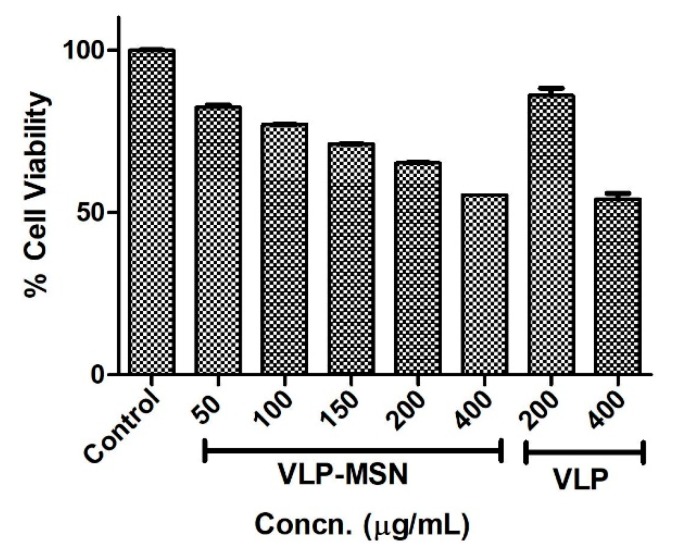
Cell viability of HEP G2 cell line after exposure to neat VLP and VLP-MSNs for 24 h. Error bars indicate the standard deviation (*n* = 3).

Drug carriers intended to be used in human must be non-toxic, biocompatible [74] and stable in living systems [75]. To determine the toxicity profile of the drug-loaded carriers, a human liver cell line (HEPG2, ATCC^®^ HB 8065^TM^) was incubated for 24 h with various concentrations of VLP-MSNs, i.e., 50, 100, 150, 200 and 400 µg/mL. After the incubation period, 10 μL of MTT reagent was added to each well and further incubated for 2 h. After this incubation time plates were read by using a bioreader at 570 nm. Our results confirmed the cytocompatibility of the drug-loaded particles. VLP-MSNs showed minimum cytotoxicity until 100 µg/mL, as shown in Figure 9. The LD_50_ of the particles was found to be 448 µg/mL. It is apparent that the viability of the exposed cell line decreased proportionally with the increased concentration of VLP-MSN, which indicated their dose-dependent behaviour. This behaviour is possibly due to exposure to higher concentrations of drug-loaded particles and direct physical contact with the cell line monolayer [45,76]. We also compared the cytotoxicity of VLP at two concentrations, namely 200 µg/mL and 400 µg/mL. The results revealed that VLP was well tolerated by the cells with more than 85% cell survival at 200 µg/mL concentration and about 55% at 400 µg/mL. Since the actual loading of VLP in MSN is far less than 200 µg/mL, therefore, it can be envisaged that the cytotoxicity of VLP is negligible, while MSNs were slightly cytotoxic yet compatible with the cells. Thus our finding further confirmed previously reported findings about the biocompatibility of silica particles [45,75,77,78]. 

### 3.10. Toxicological Evaluation

We measured various hematological (RBCs, WBCs, platelets etc.), biochemical (LFTs, RFTs, lipid profile test) parameters and body weight changes for the control and tested group before and after administration of VLP and VLP-MSNs as shown in Tables S1, S2, S3 and S4. We did not observe any significant variation in these parameters in the control and treated groups as their values remained within acceptable ranges. 

**Figure 10:**
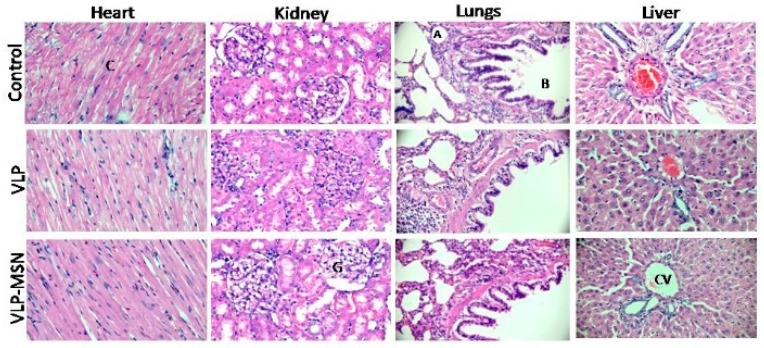
Histopathological evaluation of various vital organs i.e., heart, kidney, lung and liver of rats (At 40× objective lens in Accu-Scope 3001 Trinocular microscope fitted with a 5 megapixel camera) in control and after oral administration of VLP-MSN and VLP. CV = Central vein of liver, A = Alveolar sacs and B = bronchiole in lung, G = Glomerulus in kidney, C = Cardiomyocytes of heart.

Thus, it can be concluded that all vital organs of rats in each group remained healthy with no inflammatory response and hence, VLP and VLP-MSNs are non-toxic at the administered doses. Furthermore, all the rats remained healthy and active with only slight variations in their body weight. Additionally, histopathological images revealed no changes in vital organs namely, heart, kidney, lungs, liver in the control and treated groups (Figure 10), thus further confirming the safety of the administered formulation.

### 3.11. In-Vivo Pharmacokinetic 

*In-vivo* disposition of VLP-MSN and VLP was assessed in Sprague-Dawley rats. We determined different pharmacokinetic parameters (C_max_ (ng/mL), t_max_ (Hrs), t_1/2_ (Hrs), AUC (_0–t_) (ng/mL·h) and λ_z_ (l/h)) after oral administration of pure drug and drug-loaded particles. These parameters were calculated from the plasma of albino rats using non-compartmental pharmacokinetic analysis (Table 1).

**Figure 11:**
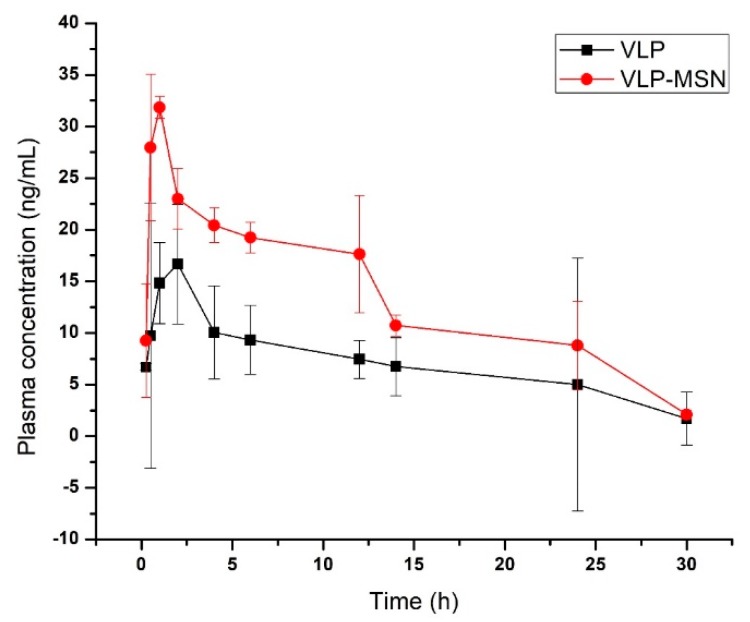
Plasma concentration–time profile of pure VLP and VLP-MSN.

C_max_ of VLP and VLP-MSN was 16.44 ± 0.48 and 31.84 ± 0.054 ng/mL, respectively, indicating approximately a two-fold increase in the bioavailability of the drug. Similarly, the AUC _0-t_ (ng/mL·h) for VLP and VLP-MSNs was 214.41 ± 1.73 and 400.50 ± 4.49, which reflects an approximately two-fold increase of the area under the curve (Figure 11). This shows that the silica particles have facilitated the dissolution of the drug in gastrointestinal (GIT) fluid and its permeability across the lumen of rats. Similar results were observed by Zhang et al. who reported a bioavailability enhancement of the poorly water soluble drug telmisartan using mesoporous silica nanoparticles [73]. Likewise in another study the authors reported a bioavailability enhancement of apigenin after development of solid dispersions of mesoporous silica nanoparticles. Apigenin-MSN solid dispersion showed an area under the curve which was 8.32 times higher compared to pure apigenin.

If we observe other parameters i.e., *T_max_* also decreased (2 to 1 h), elimination half-life (t½) for VLP-MSN decreased slightly (from 10.76 ± 0.155 to 9.20 ± 0.52 h), and the terminal elimination rate constant (λz) increased slightly (0.074 ± 0.12 to 0.079 ± 0.00041 l/h). These parameters also indicate the rapid dissolution, absorption, distribution and elimination of the drug from VLP-MSNs thus enhancing the bioavailability. Zhang et al. developed telmisartan tablets based on mesoporous micro- (MSM) and nanoparticles (MSN) and observed a decrease in *T_max_* for MSN-based tablets (0.96 h) as compared to MSM (1.42 h) and commercial (1 h) tablets, which indicates a rapid absorption from the GIT of beagle dogs [73].

Mean residence time for VLP incorporated in the formulation decreased (Table 1), which can be correlated to the *in-vivo* behaviour of MSN particles. It is reported that intact spherical MSNs are mainly accumulated in the liver after oral administration and slowly decomposed in the kidneys [79,80,81].

These results indicate a rapid release of the drug from VLP-MSNs which ultimately improves the bioavailability of the drug in rats. This rapid release of drug was due to the enhanced solubility of VLP-MSNs as compared to pure drug. These results confirmed that MSNs rapidly enhanced the solubility of VLP in GIT fluids leading to a higher concentration gradient between the lumen and blood, consequently producing a higher permeability and bioavailability of the drug after oral administration of VLP-MSNs.

## 4. Conclusions

In the present study we successfully loaded the poorly water soluble drug VLP in monodispersed silica particles having a diameter of 186 nm with good cytocompatibilty. No major interactions were observed between the drug and the silica-based carrier. The porous structure of the silica particles was confirmed by TEM and BET analysis. The pore size decreased from 5.9 to 5 nm after drug loading. VLP shows a pH dependent dissolution which could hamper its in-vivo performance after oral administration owing to fluctuations of pH throughout the GIT during fasting and feed states. These porous silica particles significantly enhanced dissolution profile of entrapped drug at higher pH values by reducing the particle size of VLP in channels, preventing crystallization, the presence of the drug in amorphous form and the large surface area of MSNs. Our in-vivo results were in line with in-vitro studies where VLP-MSNs showed rapid dissolution, absorption, higher blood concentration and possible accumulation of VLP-MSNs at the target site, i.e., the liver, as compared to pure VLP. Thus, it can be inferred that porous silica particles are non-toxic and have the potential to improve in-vitro and in-vivo performance of poorly water soluble VLP and could equally be applicable to other drugs having solubility and bioavailability-related issues.

## Figures and Tables

**Table 1:** Pharmacokinetic parameters of VLP and VLP-MSNs after single dose oral administration to rats.

Parameter	VLP	VLP-MSN
t_1/2_(h)	10.76 ± 0.155	9.20 ± 0.52
AUC _0-t_ (ng/mL*h)	214.41 ± 1.73	400.50 ± 4.49
T_max_ (h)	2	1
C_max_ (ng/mL)	16.44 ± 0.48	31.84 ± 0.054
AUC _0-inf_ (ng/mL*h)	240.61 ± 2.39	428.50 ± 4.53
λz (l/h)	0.074 ± 0.001	0.079 ± 0.00041
MRT 0-inf_obs (h)	15.87 ± 0.12	12.98 ± 0.051

## Supplementary Materials

The following are available online at https://www.mdpi.com/1999-4923/12/4/307/s1, Table S1: Biochemical blood analysis, Table S2: LFT, Table S3: RFTs and lipid profile, Table S4: Body weight (grams) changes of rats after sample administration.

## References

[1] Takagi T., Ramachandran C., Bermejo M., Yamashita S., Yu L.X., Amidon G.L. (2006). A provisional biopharmaceutical classification of the top 200 oral drug products in the United States, Great Britain, Spain, and Japan. Mol. Pharm..

[2] Kawabata Y., Wada K., Nakatani M., Yamada S., Onoue S. (2011). Formulation design for poorly water-soluble drugs based on biopharmaceutics classification system: Basic approaches and practical applications. Int. J. Pharm..

[3] Leuner C., Dressman J. (2000). Improving drug solubility for oral delivery using solid dispersions. Eur. J. Pharm. Biopharm..

[4] Bai A., Wu C., Liu X., Lv H., Xu X., Cao Y., Shang W., Hu L., Liu Y. (2018). Development of a tin oxide carrier with mesoporous structure for improving the dissolution rate and oral relative bioavailability of fenofibrate. Drug Des. Dev. Ther..

[5] De Waard H., Hinrichs W., Frijlink H. (2008). A novel bottom–up process to produce drug nanocrystals: Controlled crystallization during freeze-drying. J. Control. Release.

[6] Li W., Yang Y., Tian Y., Xu X., Chen Y., Mu L., Zhang Y., Fang L. (2011). Preparation and In Vitro/In Vivo evaluation of revaprazan hydrochloride nanosuspension. Int. J. Pharm..

[7] Wang Y., Zhang D., Liu Z., Liu G., Duan C., Jia L., Feng F., Zhang X., Shi Y., Zhang Q. (2010). In Vitro and In Vivo evaluation of silybin nanosuspensions for oral and intravenous delivery. Nanotechnology.

[8] Bali V., Ali M., Ali J. (2010). Novel nanoemulsion for minimizing variations in bioavailability of ezetimibe. J. Drug Target..

[9] Silva A., Nunes B., De Oliveira M., Koester L.S., Mayorga P., Bassani V.L., Teixeira H.F. (2009). Development of topical nanoemulsions containing the isoflavone genistein. Die Pharm. Int. J. Pharm. Sci..

[10] Tagne J.-B., Kakumanu S., Ortiz D., Shea T., Nicolosi R.J. (2008). A nanoemulsion formulation of tamoxifen increases its efficacy in a breast cancer cell line. Mol. Pharm..

[11] Khemtong C., Kessinger C.W., Ren J., Bey E.A., Yang S.-G., Guthi J.S., Boothman D.A., Sherry A.D., Gao J. (2009). In Vivo off-resonance saturation magnetic resonance imaging of αvβ3-targeted superparamagnetic nanoparticles. Cancer Res..

[12] Kim T.-H., Mount C.W., Gombotz W.R., Pun S.H. (2010). The delivery of doxorubicin to 3-D multicellular spheroids and tumors in a murine xenograft model using tumor-penetrating triblock polymeric micelles. Biomaterials.

[13] Hughes G.A. (2017). Nanostructure-mediated drug delivery. Nanomedicine in Cancer.

[14] Hans M., Lowman A. (2002). Biodegradable nanoparticles for drug delivery and targeting. Curr. Opin. Solid State Mater. Sci..

[15] Bharti C., Nagaich U., Pal A.K., Gulati N. (2015). Mesoporous silica nanoparticles in target drug delivery system: A review. Int. J. Pharm. Investig..

[16] Maggini L., Cabrera I., Ruiz-Carretero A., Prasetyanto E.A., Robinet E., De Cola L. (2016). Breakable mesoporous silica nanoparticles for targeted drug delivery. Nanoscale.

[17] Lin W., Huang Y.-W., Zhou X.-D., Ma Y. (2006). In Vitro toxicity of silica nanoparticles in human lung cancer cells. Toxicol. Appl. Pharmacol..

[18] Colilla M., González B., Vallet-Regí M. (2013). Mesoporous silica nanoparticles for the design of smart delivery nanodevices. Biomater. Sci..

[19] Lee J.E., Lee N., Kim T., Kim J., Hyeon T. (2011). Multifunctional mesoporous silica nanocomposite nanoparticles for theranostic applications. Acc. Chem. Res..

[20] Wang Y., Zhao Q., Hu Y., Sun L., Bai L., Jiang T., Wang S. (2013). Ordered nanoporous silica as carriers for improved delivery of water insoluble drugs: A comparative study between three dimensional and two dimensional macroporous silica. Int. J. Nanomed..

[21] Slowing I.I., Trewyn B.G., Giri S., Lin V.Y. (2007). Mesoporous silica nanoparticles for drug delivery and biosensing applications. Adv. Funct. Mater..

[22] Liu J., Li C., Li F. (2011). Fluorescence turn-on chemodosimeter-functionalized mesoporous silica nanoparticles and their application in cell imaging. J. Mater. Chem..

[23] Salinas A.J., Esbrit P., Vallet-Regí M. (2013). A tissue engineering approach based on the use of bioceramics for bone repair. Biomater. Sci..

[24] Ehlert N., Mueller P.P., Stieve M., Lenarz T., Behrens P. (2013). Mesoporous silica films as a novel biomaterial: Applications in the middle ear. Chem. Soc. Rev..

[25] Xu W., Riikonen J., Lehto V.-P. (2013). Mesoporous systems for poorly soluble drugs. Int. J. Pharm..

[26] Wang Y., Sun L., Jiang T., Zhang J., Zhang C., Sun C., Deng Y., Sun J., Wang S. (2014). The investigation of MCM-48-type and MCM-41-type mesoporous silica as oral solid dispersion carriers for water insoluble cilostazol. Drug Dev. Ind. Pharm..

[27] Zhang Y., Jiang T., Zhang Q., Wang S. (2010). Inclusion of telmisartan in mesocellular foam nanoparticles: Drug loading and release property. Eur. J. Pharm. Biopharm..

[28] Shen S.-C., Ng W.K., Chia L., Hu J., Tan R.B. (2011). Physical state and dissolution of ibuprofen formulated by co-spray drying with mesoporous silica: Effect of pore and particle size. Int. J. Pharm..

[29] Jia L., Shen J., Li Z., Zhang D., Zhang Q., Duan C., Liu G., Zheng D., Liu Y., Tian X. (2012). Successfully tailoring the pore size of mesoporous silica nanoparticles: Exploitation of delivery systems for poorly water-soluble drugs. Int. J. Pharm..

[30] Hu Y., Wang J., Zhi Z., Jiang T., Wang S. (2011). Facile synthesis of 3D cubic mesoporous silica microspheres with a controllable pore size and their application for improved delivery of a water-insoluble drug. J. Colloid Interface Sci..

[31] Zhu W., Wan L., Zhang C., Gao Y., Zheng X., Jiang T., Wang S. (2014). Exploitation of 3D face-centered cubic mesoporous silica as a carrier for a poorly water soluble drug: Influence of pore size on release rate. Mater. Sci. Eng. C.

[32] Kumar D., Chirravuri S.S., Shastri N.R. (2014). Impact of surface area of silica particles on dissolution rate and oral bioavailability of poorly water soluble drugs: A case study with aceclofenac. Int. J. Pharm..

[33] Martin A., García R., Karaman D.S., Rosenholm J. (2014). Polyethyleneimine-functionalized large pore ordered silica materials for poorly water-soluble drug delivery. J. Mater. Sci..

[34] Zhang Y., Zhi Z., Jiang T., Zhang J., Wang Z., Wang S. (2010). Spherical mesoporous silica nanoparticles for loading and release of the poorly water-soluble drug telmisartan. J. Control. Release.

[35] Ahern R.J., Hanrahan J.P., Tobin J.M., Ryan K.B., Crean A.M. (2013). Comparison of fenofibrate–mesoporous silica drug-loading processes for enhanced drug delivery. Eur. J. Pharm. Sci..

[36] Limnell T., Santos H.A., Mäkilä E., Heikkilä T., Salonen J., Murzin D.Y., Kumar N., Laaksonen T., Peltonen L., Hirvonen J. (2011). Drug delivery formulations of ordered and nonordered mesoporous silica: Comparison of three drug loading methods. J. Pharm. Sci..

[37] Khanfar M., Fares M.M., Qandil A.M. (2013). Mesoporous silica based macromolecules for dissolution enhancement of Irbesartan drug using pre-adjusted pH method. Microporous Mesoporous Mater..

[38] Waters L.J., Hussain T., Parkes G., Hanrahan J.P., Tobin J.M. (2013). Inclusion of fenofibrate in a series of mesoporous silicas using microwave irradiation. Eur. J. Pharm. Biopharm..

[39] Vallet-Regí M., Balas F., Arcos D. (2007). Mesoporous materials for drug delivery. Angew. Chem. Int. Ed..

[40] Cory T.J., Mu Y., Gong Y., Kodidela S., Kumar S. (2018). Sofosbuvir+ velpatasvir+ voxilaprevir for the treatment of hepatitis C infection. Expert Opin. Pharmacother..

[41] Dahan A., Miller J.M., Amidon G.L. (2009). Prediction of solubility and permeability class membership: Provisional BCS classification of the world’s top oral drugs. AAPS J..

[42] Kong F., Singh R.P. (2008). Disintegration of solid foods in human stomach. J. Food Sci..

[43] Karamanolis G., Theofanidou I., Yiasemidou M., Giannoulis E., Triantafyllou K., Ladas S.D. (2008). A Glass of Water Immediately Increases Gastric pH in Healthy Subjects. Dig. Dis. Sci..

[44] Hench L.L., West J.K. (1990). The sol-gel process. Chem. Rev..

[45] Mehmood Y., Khan I.U., Shahzad Y., Khalid S.H., Asghar S., Irfan M., Asif M., Khalid I., Yousaf A.M., Hussain T. (2019). Facile synthesis of mesoporous silica nanoparticles using modified solgel method: Optimization and In Vitro cytotoxicity studies. Pak. J. Pharm. Sci..

[46] Ganesh M., Ubaidulla U., Hemalatha P., Peng M.M., Jang H.T. (2015). Development of duloxetine hydrochloride loaded mesoporous silica nanoparticles: Characterizations and In Vitro evaluation. AAPS PharmSciTech.

[47] Zhao Z., Gao Y., Wu C., Hao Y., Zhao Y., Xu J. (2016). Development of novel core-shell dual-mesoporous silica nanoparticles for the production of high bioavailable controlled-release fenofibrate tablets. Drug Dev. Ind. Pharm..

[48] Hu L., Sun H., Zhao Q., Han N., Bai L., Wang Y., Jiang T., Wang S. (2015). Multilayer encapsulated mesoporous silica nanospheres as an oral sustained drug delivery system for the poorly water-soluble drug felodipine. Mater. Sci. Eng. C.

[49] Lee C.H., Lo L.W., Mou C.Y., Yang C.S. (2008). Synthesis and characterization of positive-charge functionalized mesoporous silica nanoparticles for oral drug delivery of an anti-inflammatory drug. Adv. Funct. Mater..

[50] Zhang M., Xu C., Wen L., Han M.K., Xiao B., Zhou J., Zhang Y., Zhang Z., Viennois E., Merlin D. (2016). A hyaluronidase-responsive nanoparticle-based drug delivery system for targeting colon cancer cells. Cancer Res..

[51] Chiang Y.-D., Lian H.-Y., Leo S.-Y., Wang S.-G., Yamauchi Y., Wu K.C.-W. (2011). Controlling particle size and structural properties of mesoporous silica nanoparticles using the Taguchi method. J. Phys. Chem. C.

[52] Morsi R.E., Mohamed R.S. (2018). Nanostructured mesoporous silica: Influence of the preparation conditions on the physical-surface properties for efficient organic dye uptake. R. Soc. Open Sci..

[53] Powers K.W., Brown S.C., Krishna V.B., Wasdo S.C., Moudgil B.M., Roberts S.M. (2006). Research strategies for safety evaluation of nanomaterials. Part VI. Characterization of nanoscale particles for toxicological evaluation. Toxicol. Sci..

[54] Masarudin M.J., Cutts S.M., Evison B.J., Phillips D.R., Pigram P.J. (2015). Factors determining the stability, size distribution, and cellular accumulation of small, monodisperse chitosan nanoparticles as candidate vectors for anticancer drug delivery: Application to the passive encapsulation of [14C]-doxorubicin. Nanotechnol. Sci. Appl..

[55] Zhang Y., Yang M., Portney N.G., Cui D., Budak G., Ozbay E., Ozkan M., Ozkan C.S. (2008). Zeta potential: A surface electrical characteristic to probe the interaction of nanoparticles with normal and cancer human breast epithelial cells. Biomed. Microdevices.

[56] Fröhlich E. (2012). The role of surface charge in cellular uptake and cytotoxicity of medical nanoparticles. Int. J. Nanomed..

[57] Clayton K.N., Salameh J.W., Wereley S.T., Kinzer-Ursem T.L. (2016). Physical characterization of nanoparticle size and surface modification using particle scattering diffusometry. Biomicrofluidics.

[58] Narayan R., Nayak U., Raichur A., Garg S. (2018). Mesoporous silica nanoparticles: A comprehensive review on synthesis and recent advances. Pharmaceutics.

[59] Sing K. (1982). Reporting physisorption data for gas/solid systems with special reference to the determination of surface area and porosity (Provisional). Pure Appl. Chem..

[60] Sevimli F., Yılmaz A. (2012). Surface functionalization of SBA-15 particles for amoxicillin delivery. Microporous Mesoporous Mater..

[61] Liu F., Wang J., Huang P., Zhang Q., Deng J., Cao Q., Jia J., Cheng J., Fang Y., Deng D.Y. (2015). Outside-in stepwise functionalization of mesoporous silica nanocarriers for matrix type sustained release of fluoroquinolone drugs. J. Mater. Chem. B.

[62] Beganskienė A., Sirutkaitis V., Kurtinaitienė M., Juškėnas R., Kareiva A. (2004). FTIR, TEM and NMR investigations of Stöber silica nanoparticles. Mater. Sci. (Medžg.).

[63] Vo C.L.-N., Park C., Lee B.-J. (2013). Current trends and future perspectives of solid dispersions containing poorly water-soluble drugs. Eur. J. Pharm. Biopharm..

[64] Sharma D., Farah K. (2018). Dynamics of induced glass transition of porous and nonporous silica nanoparticles. J. Therm. Anal. Calorim..

[65] Salonen J., Laitinen L., Kaukonen A., Tuura J., Björkqvist M., Heikkilä T., Vähä-Heikkilä K., Hirvonen J., Lehto V.-P. (2005). Mesoporous silicon microparticles for oral drug delivery: Loading and release of five model drugs. J. Control. Release.

[66] Heikkilä T., Salonen J., Tuura J., Hamdy M., Mul G., Kumar N., Salmi T., Murzin D.Y., Laitinen L., Kaukonen A. (2007). Mesoporous silica material TUD-1 as a drug delivery system. Int. J. Pharm..

[67] Maleki A., Hamidi M. (2016). Dissolution enhancement of a model poorly water-soluble drug, atorvastatin, with ordered mesoporous silica: Comparison of MSF with SBA-15 as drug carriers. Expert Opin. Drug Deliv..

[68] Hu Y., Zhi Z., Zhao Q., Wu C., Zhao P., Jiang H., Jiang T., Wang S. (2012). 3D cubic mesoporous silica microsphere as a carrier for poorly soluble drug carvedilol. Microporous Mesoporous Mater..

[69] El-Wekil M.M., Ali H.R.H., Marzouk A.A., Ali R. (2018). Enhanced dispersive solid phase extraction assisted by cloud point strategy prior to fluorometric determination of anti-hepatitis C drug velpatasvir in pharmaceutical tablets and body fluids. RSC Adv..

[70] Mehmood Y., Khan I.U., Shahzad Y., Khalid S.H., Irfan M., Asghar S., Yousaf A.M., Hussain T., Khalid I. (2019). Development and validation of a stability-Indicating RP-HPLC method for simultaneous estimation of sofosbuvir and velpatasvir in fixed dose combination tablets and plasma. Pak. J. Pharm. Sci..

[71] Mogalian E., German P., Kearney B.P., Yang C.Y., Brainard D., Link J., McNally J., Han L., Ling J., Mathias A. (2017). Preclinical Pharmacokinetics and First-in-Human Pharmacokinetics, Safety, and Tolerability of Velpatasvir, a Pangenotypic Hepatitis C Virus NS5A Inhibitor, in Healthy Subjects. Antimicrob. Agents Chemother..

[72] Charnay C., Bégu S., Tourné-Péteilh C., Nicole L., Lerner D., Devoisselle J.-M. (2004). Inclusion of ibuprofen in mesoporous templated silica: Drug loading and release property. Eur. J. Pharm. Biopharm..

[73] Zhang Y., Wang J., Bai X., Jiang T., Zhang Q., Wang S. (2012). Mesoporous silica nanoparticles for increasing the oral bioavailability and permeation of poorly water soluble drugs. Mol. Pharm..

[74] Dhanka M., Shetty C., Srivastava R. (2018). Methotrexate loaded gellan gum microparticles for drug delivery. Int. J. Biol. Macromol..

[75] Asefa T., Tao Z. (2012). Biocompatibility of Mesoporous Silica Nanoparticles. Chem. Res. Toxicol..

[76] Khan I.U., Stolch L., Serra C.A., Anton N., Akasov R., Vandamme T.F. (2015). Microfluidic conceived pH sensitive core–shell particles for dual drug delivery. Int. J. Pharm..

[77] Huang X., Li L., Liu T., Hao N., Liu H., Chen D., Tang F. (2011). The Shape Effect of Mesoporous Silica Nanoparticles on Biodistribution, Clearance, and Biocompatibility In Vivo. ACS Nano.

[78] Anirudhan T.S., Nair A.S. (2018). Temperature and ultrasound sensitive gatekeepers for the controlled release of chemotherapeutic drugs from mesoporous silica nanoparticles. J. Mater. Chem. B.

[79] Dogra P., Adolphi N.L., Wang Z., Lin Y.-S., Butler K.S., Durfee P.N., Croissant J.G., Noureddine A., Coker E.N., Bearer E.L. (2018). Establishing the effects of mesoporous silica nanoparticle properties on In Vivo disposition using imaging-based pharmacokinetics. Nat. Commun..

[80] Lee J.-A., Kim M.-K., Paek H.-J., Kim Y.-R., Kim M.-K., Lee J.-K., Jeong J., Choi S.-J. (2014). Tissue distribution and excretion kinetics of orally administered silica nanoparticles in rats. Int. J. Nanomed..

[81] Zhao Y., Wang Y., Ran F., Cui Y., Liu C., Zhao Q., Gao Y., Wang D., Wang S. (2017). A comparison between sphere and rod nanoparticles regarding their in vivo biological behavior and pharmacokinetics. Sci. Rep..

