# Supplementary material for: In-Vitro and In-Vivo Evaluation of Velpatasvir- Loaded Mesoporous Silica Scaffolds. A Prospective Carrier for Drug Bioavailability Enhancement
Source: Pharmaceutics. 2020 Mar 28;12(4):307. doi: 10.3390/pharmaceutics12040307 (PMC7238066; doi:10.3390/pharmaceutics12040307)
Supplement: Supplementary file 1 [file pharmaceutics-12-00307-s001.pdf]

Supplementary Information

In-vitro **and** ***in-vivo*** **evaluation of velpatasvir loaded mesoporous silica scaffold. A prospective carrier for drug bioavailability enhancement**

Yasir Mehmood[1], Ikram Ullah Khan[1,*], Yasser Shahzad[2*], Rizwan Ullah Khan[3], Muhammad Shahid Iqbal[4], Haseeb Ahmad Khan[5], Ikrima Khalid[1], Abid Mehmood Yousaf[2], Syed Haroon Khalid[1], Sajid Asghar[1], Muhammad Asif [6,8], Talib Hussain[2], Shefaat Ullah Shah[7]

[1]*Department of Pharmaceutics, Faculty of Pharmaceutical Sciences, Government College University Faisalabad, Faisalabad, Pakistan*

[2]*Drug Delivery Research Group, Department of Pharmacy, COMSATS University Islamabad, Lahore Campus, Lahore, Pakistan*

[3] *Department of Pathology, Prince Faisal Cancer Centre, Buraydah Al Qassim, Kingdom of Saudi Arabia*

[4]Department of Clinical Pharmacy, College of Pharmacy, Prince Sattam bin Abdulaziz University, Alkharj, Kingdom of Saudi Arabia.

[5]*Department of Pathology, FMH college of Medicine and Dentistry, Lahore, Pakistan*

[6]*Department of Pharmacology, Faculty of Pharmaceutical Sciences, Government College University Faisalabad, Faisalabad, Pakistan*

[7]*Department of Pharmaceutics, Faculty of Pharmacy, Gomal University, Dera Ismail Khan, Pakistan*

[8]*Department of Pharmacy, The Islamia University of Bahawalpur, Bahawalpur, Pakistan*

Table S1: Biochemical blood analysis

**Hematology**	**Group I (Control)**	**Group II (VLP)**	**Group III (MSN-VLP)**
**Hb (10-15) g/dL**	12.2	11.8	12.8
**Hct**	41.8	43.6	39.7
**WBCs × 10^9 /l**	5.2	6.5	6.2
**RBCs × 10^6/mm^3**	5.76	5.72	5.26
**Platelets × 10^9/l**	286	258	257
**Monocytes (%)**	07	06	03
**Neutrophils (%)**	40	40	35

**Lymphocytes (%)**	50	52	60
**MCV (%)**	72.1	74.2	73.2
**MCH pg/cell**	18.6	21.8	21.4
**MCHC (%)**	30.4	30.4	30.1
**Esinophils**	03	02	02
**Blood Sugar Random mg/dl**	52	71	62
**Uric Acid (Serum)mg/dl**	5.76	8.2	7.1

Table S2: LFT

**Liver Function Tests**	**Group I (Control)**	**Group II (VLP)**	**Group III (VLP-MSN)**
**Bilirubin Total mg/dL**	0.7	1.0	0.7
**Conjugated mg/dL**	0.2	0.2	0.2
**S.G.P.T U/L**	68	86	74
**S.G.O.T U/L**	59	74	68
**Alkaline Phosphatase U/L**	102	121	129
**Total Protein G/dl**	9.2	8.7	9.1
**Albumin G/dl**	3.0	3.2	3.0
**Globulins G/dl**	6.2	5.5	6.1

**A/G Ratio**	0.5	0.6	0.5

Table S3: RFTs and lipid profile

**Biochemical analysis**	**Group I (Control)**	**Group II (VLP)**	**Group III (VLP-MSN)**
**Creatinine (0.8-1.8) (mg/dl)**	0.9	1.1	1.0
**Urea (mmol/l)**	72	86	76
**Uric acid (mg/dl)**	7.6	8.2	7.1
**Cholesterol (10-80) (mg/dl)**	138	131	126
**Triglycerides (46-68) (mg/dl)**	52	48	48

Table S4: Body weight (grams) changes of rats after sample administration

		**1ST Day**	**5TH Day**	**10th Day**	**14th Day**
**Control**	**Mean**	206.3	211.67	215	218.67
	**SD**	11.8	12.6	13.2	14.1
**VLP RAW**	**Mean**	205.33	209.67	215.33	216.33
	**SD**	8.4	10.01	14.6	16.9
**VLP MSN**	**Mean**	209.67	213.33	215	217.33
	**SD**	10.0	12.6	14.5	16.6
